# Self-Assembled PVP-Gd Composite Nanosheets via Ultrasound Synthesis for Targeted Acrylamide Sensing in Food Safety

**DOI:** 10.1021/acs.jafc.4c08460

**Published:** 2025-02-11

**Authors:** Sahar Pakbaten Toopkanloo, Hui-Fen Wu

**Affiliations:** † International PhD Program for Science, National Sun Yat-Sen University, Kaohsiung 80424, Taiwan; ‡ Department of Chemistry, National Sun Yat-Sen University, Kaohsiung 80424, Taiwan; § School of Pharmacy, College of Pharmacy, Kaohsiung Medical University, Kaohsiung 807, Taiwan; ∥ School of Medicine, College of Medicine, National Sun Yat-Sen University, Kaohsiung 80424, Taiwan; ⊥ Institute of Medical Science and Technology, National Sun Yat-Sen University, Kaohsiung 80424, Taiwan; # Institute of Precision Medicine, National Sun Yat-Sen University, Kaohsiung 80424, Taiwan; ∇ Institute of BioPharmaceutical Science, National Sun Yat-Sen University, Kaohsiung 80424, Taiwan

**Keywords:** 2D nanosheets, PVP-Gd composite, selective sensor, fluorescence quenching, acrylamide detection

## Abstract

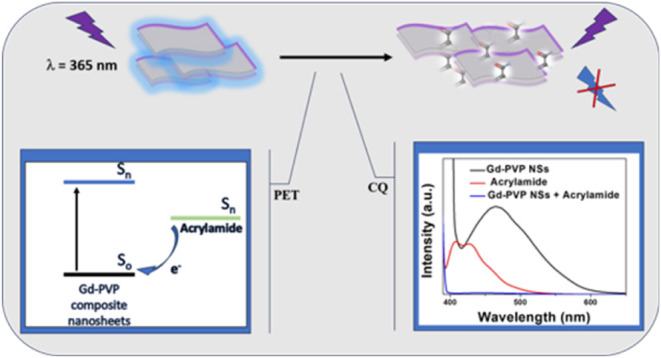

Acrylamide (AM) is a recognized carcinogen and neurotoxin, posing a significant threat to food safety and human health. Therefore, developing sensitive and convenient methods for AM detection in food samples is essential. This study responds to the urgent need for sensitive and selective detection of AM, a hidden hazard in food, to safeguard public health and environmental safety. We present the development of a novel two-dimensional ultrasound-synthesized PVP-Gd composite nanosheet platform for precise AM sensing. These self-assembled nanosheets, constructed from gadolinium (Gd) and poly(vinylpyrrolidone) (PVP), exhibit remarkable stability and robust blue fluorescence, with a quantum yield of 45.01% upon excitation at 380 nm. A full factorial design was employed to optimize the synthesis process, revealing significant parameter interactions. The optimized nanosheets demonstrated a strong quenching effect upon acrylamide exposure, resulting in a high-performance acrylamide sensor with an impressively low detection limit (9.4 nM) and a broad linear response range. This innovative sensor platform offers a promising approach for environmental monitoring and food safety applications, effectively addressing the risks associated with acrylamide.

## 1 Introduction

Acrylamide (IUPAC name, 2-propanamide, C_3_H_5_NO, AM) is a water-soluble, odorless, crystalline solid initially created for commercial use. It is a highly reactive compound found naturally in tobacco smoke and starchy foods cooked at high temperatures. Additionally, it is commonly used in food, cosmetics, plastics manufacturing, and water treatment as a flocculant.^1,2^ AM enters the body through inhalation, ingestion, and other routes, prompting global concerns due to its widespread presence and potential cancer-causing effects, backed by numerous studies. Exposure to AM is associated with elevated inflammatory markers, indicating its impact on inflammatory responses. Research indicates significant risks of neurotoxicity, reproductive problems, and cancer linked to AM exposure.^3^ Absorption in the digestive tract raises concerns about genetic damage and mutations, leading to serious health issues. Classified as a “probable human carcinogen” and a “Substance of Very High Concern” by key agencies, identifying and measuring AM is crucial for ensuring food and environmental safety.^3,4^

Current methods for AM detection often face limitations such as time-consuming procedures, high costs, and reliance on specialized equipment, hindering effective food safety monitoring. Conventional techniques like high-performance liquid chromatography (HPLC), gas chromatography (GC), size exclusion chromatography, liquid chromatography–mass spectrometry (LC-MS), and gas chromatograph–mass spectrometry (GC-MS) are complex, require extensive training, and often necessitate large sample sizes. GC-based methods are further complicated by AM’s low vapor pressure, requiring derivatization. While effective, biobased methods like enzyme-linked immunosorbent assays (ELISA) and aptamer-based sensors suffer from limitations in antibody availability and complex procedures. ELISA methods, such as those described by Preston et al.^5^ and Franek et al.,^6^ and aptamer-based sensors, as explored by Khoshbin et al.,^7^ often involve intricate procedures and specialized reagents. A major challenge with these biosensors lies in the limited availability and low production of specific antibodies in serum.^1^ This limitation stems from AM’s small molecular weight and low immunogenicity, which hinders the exposure of its epitopes and makes it difficult to generate high-affinity antibodies for sensitive detection. Piezoelectric biosensors, which rely on piezoelectric crystals with opposite charges and specific binding sites for acrylamide, have also been investigated for food sample analysis. However, these methods are hindered by complex supramolecular preparation, the need for skilled operators, and costly instrumentation.^1^ As a result, their application in routine food science analysis for acrylamide detection has been limited.

Nanoanalytical fluorescent sensor platforms offer a promising solution, leveraging materials that change optical properties in response to chemical changes. These sensors are renowned for their high affinity, ease of synthesis, and direct interaction mechanism, enabling exceptional sensitivity and selectivity for AM detection. As reported by Han et al.,^8^ fluorescence sensors are generally simpler to fabricate, more cost-effective, and offer faster response times due to their direct optical readouts. Beyond that, they provide high sensitivity and selectivity thanks to efficient “turn-on” or “turn-off” mechanisms and strong molecular interactions. Fluorescence sensors directly detect target analytes through light emission, offering higher sensitivity than many indirect detection methods, such as electrochemical sensors, due to their ability to amplify fluorescence signals and carefully designed fluorophores and binding sites.^8,9^ This design enhances selectivity by reducing interference from nontarget analytes and provides rapid response times essential for real-time monitoring.^10^ Unlike the complex multistep synthesis required for solid-state upconversion sensors and the specialized equipment needed for quartz crystal microbalance techniques, fluorescence methods provide a more accessible and user-friendly approach to nanomaterial-based sensing.^8−10^ In studies reported by Han et al.^11^ and Yin et al.,^12^ the fluorescence sensing platform successfully demonstrated its ability to rapidly and sensitively discriminate and assess foodborne pathogens.

Composite nanoparticle sensors, often referred to as nanocomposite sensors, include organic–inorganic nanocomposites (OINCs) or hybrids typically consisting of two nanoscale components, A and B. In most cases, component A comprises organic dyes either encapsulated within (hybrid) or attached to component B (composite), an inorganic matrix.^13,14^ Indeed, integrating nanoscale components into these systems brings about significant drawbacks, encompassing challenges related to stability in harsh environments, potential toxicity leading to bioaccumulation, and the requirement for effective methods for regeneration and reuse.^15,16^ Additionally, the use of fluorescent agents like toxic organic dyes and hazardous modification steps, particularly those involving heavy metal-based quantum dots (QDs),^17^ as opposed to emerging biocompatible alternatives like carbon dots (CDs),^18^ raises further concerns.^11^ Polymer-derived nanocomposites find broad applications in biomedicine and environmental science, owing to their outstanding biocompatibility, efficient degradability, precise self-assembly control, and unique biomimetic properties.^13,19,20^ Poly(*N*-vinyl-2-pyrrolidone) (PVP; (C_6_H_9_ON)*_n_*) is a safe, antifouling polymer widely used in various industries, including food, medicine, and pharmaceuticals.^21^ Its nontoxic nature enables oral administration, making it suitable for various human and animal applications in these fields.^22−26^ In nanotechnology, PVP is a versatile surface modifier, functioning as both a surfactant and structural director in nanoparticle synthesis.^27^ It effectively manages particle shape and size while stabilizing the nanoparticles, aided by its nontoxicity, high biocompatibility, and excellent wetting properties.^28^ The hydrophilic nature of PVP, combined with hydrophobic interactions from its carbon chain, facilitates even nanoparticle dispersion.^29^ Its polar structure, amphiphilic properties, and proton-accepting capabilities further expand its adaptability, supporting its use in applications such as fluorescent probes. Zhang et al.^27^ developed a fluorescence sensor for the detection of zearalenone, a mycotoxin that poses serious risks to food safety and human health, using a poly(vinylpyrrolidone)-modified Zr-MOF (PVP-UiO-67). The addition of PVP to UiO-67 significantly enhanced the material’s water dispersibility and stability. The sensor exhibited selective and sensitive fluorescence quenching in the presence of zearalenone, with a detection limit as low as 7.44 nM.

The lanthanides, known as the rare-earth elements, are large atoms with coordination numbers varying from 7 to 14. Rare-earth oxides represent advanced materials commonly utilized as host lattices for developing sensors and luminescent substances.^30,31^ They are renowned for their exceptional chemical and thermal stability. Gadolinium oxide (Gd_2_O_3_) stands out as a particularly promising material for creating contrast agents in applications involving magnetic resonance and fluorescence imaging.^32^ Furthermore, the intrinsic optical properties of Gd_2_O_3_ allow it to exhibit sharp wavelength absorptions and photostability, making it highly valuable for imaging applications. The incorporation of extra lanthanide ions into the matrix is a commonly used approach. On the other hand, the improvement of optical properties in Gd-based materials through cross-linking with a polymer is a well-established and highly efficient process, yet it introduces a brand-new dimension to the field. This strategic choice of polymers empowers the development of photoluminescent materials characterized by remarkable features, such as substantial Stokes shifts, precisely defined emission spectra (spanning the visible or near-infrared regions), prolonged lifetimes, reduced photobleaching, and the capability for multiphoton absorption.^33^ The large size of Gd ions can offer more surface area for interaction with surrounding molecules like PVP. This allows for more potential binding sites for PVP to interact with the Gd oxide surface. Besides, the ability of the Gd atoms to adopt different coordination numbers provides some flexibility in how they interact with ligands. This can help accommodate the binding geometry of PVP molecules. Additionally, it is possible to fine-tune the excitation and emission wavelengths as needed when considering their interaction with organic compounds like PVP. In a study led by Premlatha et al.,^34^ a Co–Gd_2_O_3_ nanocomposite was synthesized via electrodeposition as a means of detecting l-cystine. Their findings suggest that the Gd_2_O_3_ nanoparticles enhance electron transfer, leading to more electrochemically active cobalt species due to additional anchoring sites. Owing to these distinctive properties, Gd lattices are considered excellent materials for a wide array of photoluminescence applications, encompassing not only biological domains but also the environmental field.

The study aims to develop an advanced organic–inorganic nanocomposite sensor by introducing a novel method for constructing a photostable two-dimensional (2D) metal–polymer composite nanosheet probe. This sensor is specifically designed for the effective detection of acrylamide (AM), a known human carcinogen. Utilizing a low-intensity focused ultrasound (LIFU)-assisted solvothermal synthesis approach, the study focuses on designing a finely tuned and optimized formulation of this 2D metal–polymer nanosheet, enhancing its sensitivity and stability for reliable AM detection.

## 2 Materials and Methods

### 2.1 Reagents and Materials

Gadolinium(III) oxide (Gd_2_O_3_), sodium borohydride (NaBH_4_), and poly *N*-vinylpyrrolidone (PVP) were provided by Sigma-Aldrich. Acrylamide (AM, C_3_H_5_NO, 99.0%), along with interfering substances such as acrylic acid (C_3_H_4_O_2_, 99.0%), asparagine (C_4_H8N_2_O_3_, 99.0%), ascorbic acid (C_6_H_8_O_6_, 99.0%), glucose (C_6_H_12_O_6_, 99.0%), succinic acid (C_4_H_6_O_4_, 99.0%), and acetic acid (CH_3_COOH, 99.0%) were supplied from Thermo Fisher Scientific Inc. Ethyl alcohol (C_2_H_6_O, 99.8%) and deionized water (DI H_2_O, resistivity ∼18.2 MΩ·cm) were obtained from Thermo Fisher Scientific Inc. Dilute working solutions of lower concentrations were freshly prepared each day by mixing the stock solution with double distilled water. All chemicals employed in this process were of analytical grade.

### 2.2 Material Characterization

The spectral data were collected using various instruments. Ultraviolet–visible (UV–vis) spectra were recorded on an Evolution 201 UV–visible spectrophotometer from Thermo Scientific. Photoluminescence (PL) spectra were acquired using a Hitachi Fluorescence Spectrophotometer F-2700 (Hitachi, Japan). High-resolution transmission electron microscopy (HRTEM) images and selected area electron diffraction (SAED) patterns were captured with an electron microscope, specifically the JEOL JEM2100 (Japan), operating at 200 kV. Particle size and ζ-potential (ZP) analyses were conducted using an ELSZ-2000 dynamic light scattering (DLS) instrument from Otsuka Electronic (Japan). X-ray photoelectron spectroscopy (XPS) analysis was conducted using an Auger electron spectrometer (JEOL, Japan). Raman measurements were executed using a laser with a wavelength (λ) of 633 nm from JOBIN-YVON T64000. The self-assembled samples were deposited onto a glass substrate for Raman measurements.

### 2.3 Synthetic Procedure of 2D PVP-Gd Composite NSs

The preparation process involved two steps, outlined below.

**Step 1**: Formulation of PVP dispersions with different concentrations:

Our primary focus was to ascertain the optimal polymer concentration that would yield the desired metal–polymer composite nanosheets with maximum efficiency and effectiveness. We meticulously carried out experiments, investigating the impact of two different PVP concentrations (*X*_1_): 3 and 6% (wt) employing a general full factorial design approach (Table S1). Initially, PVP was dissolved in a mixture of ethanol and water with a 1:1 mass ratio, yielding solutions at the specified concentrations. The mixture was then gently stirred at room temperature for 10 min, resulting in a transparent solution. Following this, the solution underwent bath sonication at 45 °C for 30 min, ultimately yielding a hydrophilic supramolecular dispersion.

**Figure 1:**
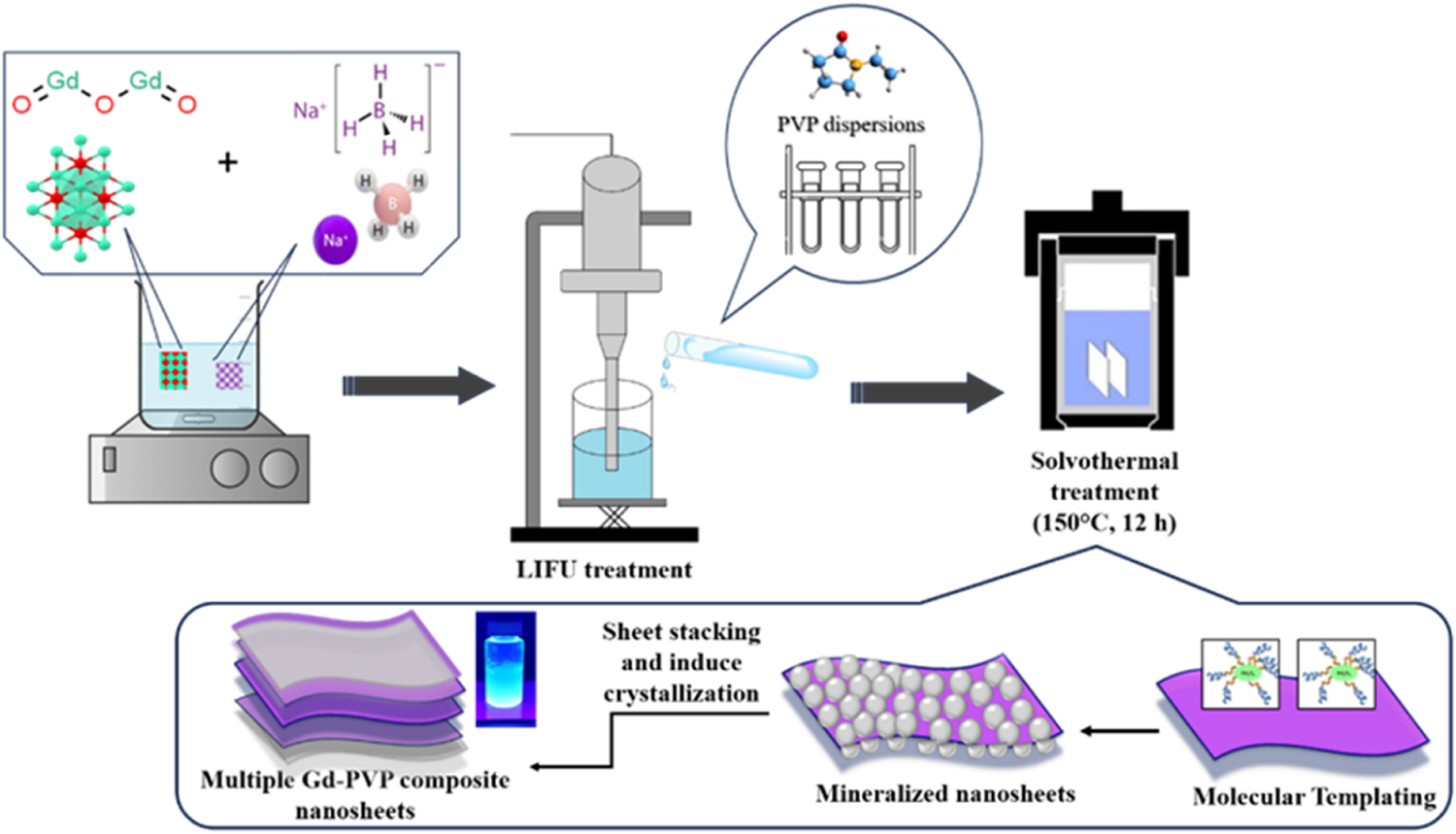
Schematic illustration for the synthesis of 2D PVP-Gd composite nanosheets.

**Step 2**: LIFU-assisted solvothermal fabrication of PVP-based Gd composite nanosheets:

In this step, we aimed to effectively engineer functionalized Gd composite nanosheets based on PVP using a tailored synergistic approach. This approach was aided by low-intensity focused ultrasound (LIFU, <3W/cm^2^) and included the incorporation of a solvothermal process (see Figure 1). Two different PVP concentration solutions (as detailed in Section 2.3, step 1) were used, in combination with the presence or absence of the reducing agent (*X*_2_, NaBH_4_) and two different LIFU durations (*X*_3_) of low (10 min) and high (30 min). This variation aimed to achieve optimal composite nanosheet assemblies. The entire procedure was repeated 6 times following a 6-run design, as detailed in Table S1.

The procedure began by preparing a solution, dissolving 1.5 g of Gd_2_O_3_ in absolute ethyl alcohol. Concurrently, 1 g of NaBH_4_ (for F2, F4, and F5) was gradually added to the solution while maintaining a temperature below 20 °C. The mixture was stirred at 35 °C for 10 min, forming a milky white suspension. Following this, the suspension underwent continuous-mode LIFU treatment for durations of 10 and/or 30 min, as specified by the treatment design. The dispersion temperature was monitored throughout the LIFU treatment, and the temperature only rose by 4 °C, going from room temperature (25 °C) to 29 °C. This step aimed to achieve consistent dispersibility, ensure a uniform particle size distribution, increase reaction yield, and reduce the reaction time to facilitate molecular assembly between the polymer and metal molecules. While the ultrasonication was in progress, the as-prepared PVP suspension solutions (6 mL) were separately and evenly dropped into the solution. The resulting cloudy solution was then transferred to a Teflon-lined stainless autoclave for supercritical seeding, where it was exposed to a temperature of 150 °C for 12 h. The obtained residue was subsequently rinsed with water and subjected to centrifugation at 10,000 rpm for 10 min to eliminate any remaining reactants. Lastly, the washed product was dried in a vacuum oven at 80 °C for 2 h, completing the drying process and resulting in the formation of crystalline two-dimensional functionalized Gd composites-based PVP. After cooling the reaction solution to room temperature (25 °C), it was diluted 100-fold with deionized water and then stored as the 2D PVP-Gd composite NSs stock solution in the dark. The optimization process to achieve optimal nanosheet assemblies involved evaluating the %particle size increase, ζ-potential, %reaction yield, and %quantum yield of the prepared nanocomposites.

The yield of 2D PVP-Gd composite NSs was calculated using the following equation (eq 1)
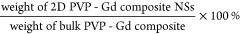
1Here, the “Weight of 2D PVP-Gd composite NSs represents the mass of the resulting nanoscale product, and the “Weight of bulk PVP-Gd composite” refers to the initial mass of the precursor solution before undergoing ultrasonic irradiation. The calculated yield provides the percentage of 2D PVP-Gd composite NSs obtained from the starting material.

### 2.4 Development of 2D PVP-Gd Composite NSs Fluorescence Probe

The quantity of material incorporation, incubation time, and incubation temperature were optimized. First, 10 mg of the nanosheet powder was dissolved in 10 mL of deionized water (pH = 7.2) and subsequently diluted to achieve a concentration of 10 μg/mL. The solution underwent incubation at room temperature (25 °C) for 45 min, forming the fluorescence probe. Following this, fluorescence spectra were recorded within the 300–400 nm range with 10 nm increments, and the excitation intensity was optimized for subsequent experiments. The measurements were carried out with slit widths of 5.0 nm and 10.0 nm for excitation and emission, respectively. The optimized conditions for constructing 2D PVP-Gd composite NSs were consistent with those employed in the experimental procedure.

### 2.5 Procedure of Acrylamide (AM) Detecting

In a 5 mL centrifuge tube, 1 mL of 2D PVP-Gd composite NSs stock solution was introduced to 1 mL AM solution in serial concentration (0.1–50 μmol/mL). After a 2 min reaction duration, the mixture was thoroughly mixed to obtain fluorescence absorption data. The fluorescence intensity of the above solutions was detected at λ_ex_ = 380 nm (optimized nanosheets sample). A function formula relating AM concentration (*X*) and ΔFluorescence intensity (*Y*) was established. The detection limit (LOD) was calculated using the formula LOD = 3σ/*K*, where σ represents the standard deviation of 10 blank samples, and *K* is the slope of the function formula. To evaluate selectivity, experiments were conducted by substituting solutions of potential interfering substances into the 2D PVP-Gd composite NSs suspension instead of AM, using the same procedure as described above. The fluorescence intensity was subsequently compared with the activity of AM, and a calibration plot was generated to illustrate the relationship between these two parameters.

### 2.6 Quantum Yield Determination

The quantum yield of the freshly synthesized 2D PVP-Gd composite NSs was assessed using quinine sulfate in 0.1 M H_2_SO_4_ (with a known quantum yield of 0.54 in water) as the standard sample. The quantum yields of fluorescent substances were computed employing the following equation (eq 2)
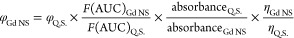
2In the equation: φ denotes the quantum yield, Q.S. refers to quinine sulfate (used as a reference), *F*(AUC) represents the fluorescence area under the curve, absorbance signifies the absorbance at 370 nm, and η stands for the solvent refractive index of the sample (in this case, ethyl alcohol: 1.3614).

### 2.7 Stability Study

To evaluate the long-term stability of the composite nanosheets, we monitored particle size variations using DLS over 30 days. Variations in particle size over time can indicate alterations in the composite nanosheets. Changes in size may reflect modifications in surface properties, such as surface charge, chemical composition, or the formation of aggregates. Accordingly, particle size and particle size stability measurements^35^ were performed to assess the stability of the nanoparticles (refer to S1).

The polymer matrix confers stability upon the optimized nanosheets, ensuring their structural integrity persists amidst diverse environmental conditions, thereby upholding the sensor’s efficacy over time. In order to characterize the stability of the prepared probe, a PL test was utilized to estimate the durability of the developed fluorescent nanosheets. Photostability was assessed by recording the initial PL emission spectra of the fluorescent samples at the respective collected excitation wavelengths. Samples were then exposed to continuous UV illumination (365 nm) using a high-intensity UV lamp for 1 h. During the exposure period, PL emission spectra were measured at 10 min intervals to monitor any changes in fluorescence intensity. Control samples that were not subjected to continuous illumination were included to account for any changes due to environmental factors. Data analysis involved comparing the PL emission spectra over time to assess photostability. Stable fluorescence intensity over time indicated good photostability, whereas a significant decrease suggested photobleaching.

To further assess the stability of the optimized PVP-Gd composite nanosheets (F4), a temperature-dependent photoluminescence (PL) assay was conducted. The assay involved recording the PL emission of F4 under varying temperatures to assess any thermal stability impacts on the composite’s luminescent properties.

For this purpose, F4 was excited at a wavelength of 380 nm, and PL emission spectra were recorded at temperatures ranging from room temperature (25 °C) up to 70 °C. Each temperature was held constant until the PL signal stabilized to ensure accurate measurement. The emission data were then analyzed to evaluate the thermal stability and integrity of the PVP-Gd composite nanosheets under these conditions.

### 2.8 Analysis of AM in the Real Samples

Potato chips and biscuits were sourced from a local market in Kaohsiung City, Taiwan. A sample weighing 5.0 g was homogenized with 20.0 mL of water for 5 min and then centrifuged for 10 min at 8000 rpm in 50 mL centrifuge tubes. Afterward, a defatting step was performed using 20 mL of *n*-hexane. The resulting sample extract was then diluted 50-fold with distilled water to minimize any potential sample matrix effects. The diluted extract from the biscuit sample was subjected to the same derivatization and assay procedures as described earlier. The calibration plot was established using the real sample matrix, with three independent replicates at each calibration level to ensure reliable and reproducible results. To assess whether the matrix significantly impacted the calibration, a statistical comparison between the standard calibration and matrix calibration was performed using an independent *t* test. The *p*-value obtained was 0.05, indicating a significant difference between the two calibrations and suggesting the presence of matrix interference.

### 2.9 Experimental Design and Statistical Analysis

To optimize the preparation process, we employed a 3-factor, 2-level full factorial design. This design evaluated the effects of three independent variables

- Mass ratio of PVP (*X*_1_)
- Presence/absence of NaBH_4_ (*X*_2_) (coded as 0 for absence and 1 for presence)
- Ultrasound duration time (*X*_3_)

The dependent variables measured were

- ζ-potential (ZP) (*Y*_1_)
- % particle size increase (*Y*_2_)
- % reaction yield (*Y*_3_)
- % quantum yield (*Y*_4_)

Statistical analysis of the factorial design preparation was conducted using Minitab statistics software 19 (Minitab Inc., State College, PA). Significance was determined at *p* < 0.05, employing Tukey’s test. Subsequently, optimization was executed to identify the most favorable formulation conditions, leading to the optimal composite nanosheet formulation. Fluorescence and Raman spectra were acquired using OriginPro software (Origin Lab, MA). Graphs, including standard curves, were generated using Microsoft Excel 2021, version 16 (Microsoft Corporation, WA). All reported values represent the means ± standard deviation (SD) of two independent experiments, with measurements performed in triplicate.

## 3 Results and Discussion

### 3.1 Optimizing the Fabrication Process of 2D PVP-Gd Composite Nanosheets

**Figure 2:**
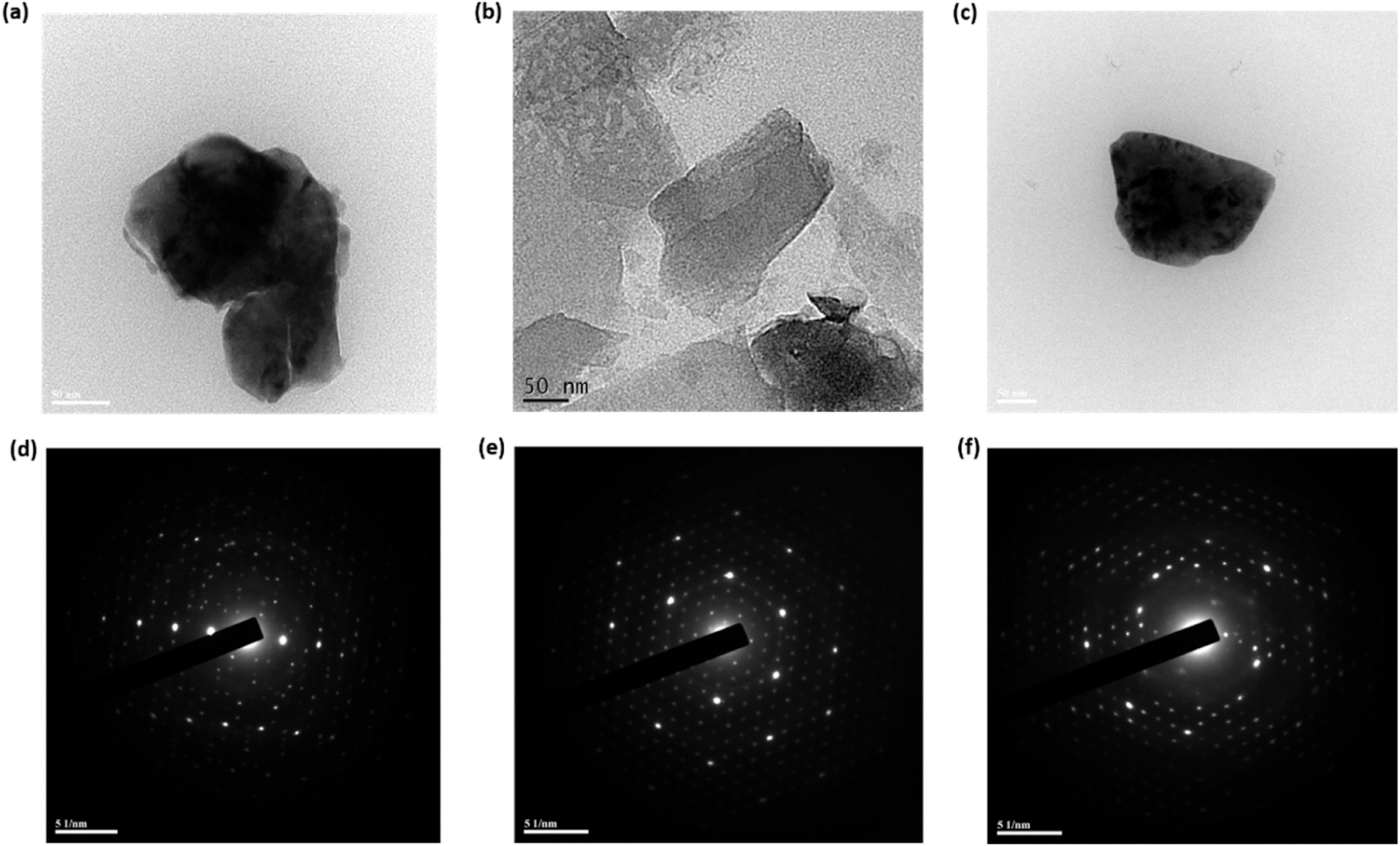
(a–c) TEM images (50 nm scale) and (d–f) SAED patterns of PVP-Gd composite nanosheets for formulations F2, F4, and F5, respectively.

This study identified the optimal preparation conditions for 2D Gd-PVP composite NSs using the LIFU-assisted solvothermal method. Combining PVP polymer with Gd, a ductile rare-earth element, in ethanol under reduction with NaBH_4_ yielded two-dimensional network metal–polymer composites for samples F2, F4, and F5 (Figure 2). Samples F1, F3, and F6 exhibited neither fluorescence nor well-defined nanosheet morphology (Table S2 and Figure S1). Selected through comparison with control experiments, PVP demonstrated favorable self-assembly capabilities, forming uniform-sized 2D PVP-Gd composite NSs. The observed values continued to increase with higher PVP concentration and reduced ultrasonication time for the analyzed samples, arranged in the following sequence: F4 > F5 > F2 > F6_nf_ > F3_nf_ > F1_nf_ (where “nf” denotes nonfluorescent). The DLS confirmed all fabricated composite systems were within the nanoscale range, with initial sizes varying between 200 and 800 nm. However, after 24 h, only formulations F4, F5, and to a lesser extent F2, maintained uniform particle distribution and exhibited a narrower size range, averaging approximately 400 nm in diameter (data not shown). Furthermore, all developed formulations displayed a negative charge ranging from ∼+7 to ∼+29 mV (Table S3). As the concentration of PVP in the mixture decreased, the ZP became less negative, a change found to be statistically significant (*p* < 0.05). The improvement in ZP values was observed at a 6 wt % PVP concentration may be attributed to reduced system heterogeneity and molecular re-engineering of the nanosheets. This is supported by enhancements in both %particle size stability and the percentage of reaction yield (Table S3). The increase in ultrasonication time negatively influenced the surface charge of the PVP-Gd composites (*p* < 0.05). This is suggested that prolonged continuous-mode LIFU treatment might lead to excessive agitation and fragmentation of particles, resulting in decreased stability of the resultant system and a reduction in surface charge.^36^ Not only this, extended ultrasonication might induce undesirable chemical or physical alterations on the surface properties of the assembling PVP-Gd composites.^37^ This could result in the disruption of existing coordination interactions between Gd ions and PVP atoms, leading to alterations in the surface charge characteristics of the final products. In contrast, a 10 min LIFU treatment is expected to enhance mixing and mass transfer (refer to %reaction yield results), potentially facilitating deaggregation and deagglomeration to achieve a more uniform suspension.

TEM was used to characterize the morphology of the as-prepared nanocomposites. Figure 2a–f reveals the influence of variations in PVP concentration and the LIFU exposure time on the self-assembly and crystallization of PVP molecules on the Gd surface, forming different nanocomposite morphologies. The results indicate that the composite F4 (Figure 2b) displayed consistent thickness, offering a substantial support area for composite preparation, possibly attributed to the ongoing epitaxial growth of both Gd and PVP. These results indicate that the PVP molecule is softly attached to the Gd atom and the charge transfer (CT) from PVP to Gd takes place.

Gd and PVP were effectively integrated within the matrix, with PVP atoms uniformly distributed on the surfaces of the prepared nanosheets, forming a single layer of nanosheets. TEM images of formulations F2 and F5 (Figure 2a,c) do not exhibit the perfect nanosheet morphology observed in F4; instead, they display denser and more uneven structures, with F2 showing additional edge irregularities.

TEM images of formulations F1_nf_, F3_nf_, and F6_nf_ (Figure S1a–c) reveal substantial heterogeneity with agglomerates, suggesting the formation of misshapen and nonuniform structure that deviates significantly from the intended nanosheet morphology. This is further supported by the broad size distributions, low colloidal stabilities, and poor reaction yields. These observations likely stem from the absence of NaBH_4_, a reducing agent, in these formulations, hindering the formation of well-defined nanosheets. In addition, lowering the PVP content to 3% (formulation F1) and prolonging the sonication process (formulation F3) resulted in the formation of even bulkier and less stable structures.

The crystalline structure is further confirmed by the selected area electron diffraction (SAED) pattern, which matches theoretical predictions (Figure 2d–f). The energy dispersive X-ray spectrometer (EDX) data (Figure S2) confirms the presence of only the expected elements: Gadolinium (Gd), Carbon (C), Oxygen (O), and Nitrogen (N). Both elements are inherent to the PVP structure, as PVP’s molecular formula (C_6_H_9_NO)_n_ contains both N and C in its backbone. The nitrogen and carbon signals observed in the elemental mapping strongly suggest that PVP is incorporated within the composite structure. This supports our assertion that PVP is acting as a stabilizing and binding agent around Gd, creating the composite. The results further indicate the absence of any unwanted impurities in the 2D PVP-Gd composite NSs (F4). It is important to note that EDX spectra were taken from various locations on the samples, consistently demonstrating the uniform distribution of elements throughout the material. The uniform distribution of elements throughout the composite is particularly noteworthy. The elemental mapping (Figure S3) clearly shows that Gd, C, O, and N are evenly dispersed across different areas of the nanosheets, indicating that PVP effectively homogeneously stabilizes the Gd particles. This distributed mapping suggests that PVP is not merely confined to the surface but is intercalated within the composite, contributing to its structural integrity and stability. The consistency of the elemental signals from multiple locations further reinforces the uniformity of the composite material, which is crucial for its potential application as a reliable sensor.

Hence, a likely scenario for the interaction of Gd with PVP might involve alternative interactions aimed at inducing Gd–O–PVP linkage, where an oxygen atom from a carbonyl group (C=O) in the PVP molecule interacts with Gd ions on the surface of the oxide. These interactions could lead to the formation of a PVP-coated Gd oxide with good dispersion and stability, contributing to the creation of a well-defined, extended network characteristic of a metal–polymer composite. It is important to note that while the Gd–O–PVP linkage is a key factor, other interactions might also contribute to the overall binding between Gd and PVP.

The aforementioned results indicate that the presence of small organic molecules (PVP) in the solution is attributed to the formation of 2D nanosheet structure, wherein they play a crucial role in regulating the crystal morphology and synthesis rate of the resulting nanosheets by restricting crystal growth, as supported by literature evidence.^38,39^

Our findings demonstrate that a 10 min ultrasonic treatment effectively disperses precursor materials without compromising the metal–polymer hydrogen bonds. This leads to a more uniform reaction environment, crucial for optimal product quality. Extending the treatment to 30 min appears detrimental, causing excessive mixing and nanoparticle aggregation. Additionally, increasing the polymer concentration can improve the balance between efficient exfoliation and precursor mixing.^40^ By introducing Gd_2_O_3_ as a precursor, the abundant amount of PVP could participate in the precipitation or self-assembly process, directing the formation of thin, sheet-like structures during the reaction. The specific interactions between a sufficient number of PVP molecules and the Gd precursor would determine the final morphology of the nanosheets. Not only that, but the Gd oxide, presented in a layered form similar to certain types of rare earth hydroxides, may induce PVP to act as an intercalating agent. This entails its gentle insertion between the layers (using a 10 min ultrasound), potentially causing swelling and exfoliation of the layers, thereby leading to the formation of individual nanosheets.^41^ This ultimately promotes the formation of 2D PVP-Gd composite nanosheets with desirable properties and facilitates complete synthesis, as evidenced by the 92% reaction yield observed in the composite F4.

### 3.2 Optical Properties of the Prepared PVP-Gd Composites

PL measurements were performed to determine the fluorescence properties of the synthesized formulations. Results indicated that samples F1, F3, and F6 exhibited no fluorescence, denoted as F1_nf_, F3_nf_, and F6_nf_, consistent with their excitation wavelengths (λ_ex_) below 300 nm. In contrast, samples F2, F4, and F5 displayed fluorescence. To further investigate the influence of excitation wavelength on these fluorescent samples (F2, F4, and F5), PL emission spectra were collected at various excitation wavelengths ranging from λ_ex_ = 300 to 400 nm in 10 nm intervals (Figure S4a–c). Analysis of the results revealed a red shift in F4 (λ_ex_ = 380 nm), whereas F2 (λ_ex_ = 320 nm) and F5 (λ_ex_ = 310 nm) displayed a blue shift. Sample F4 displayed an enhanced emission peak at 460 nm upon excitation at 380 nm, affirming λ_ex_ = 380 nm as the optimal excitation wavelength for measuring emission spectra (Table S2).

The lack of fluorescence in samples F1, F3, and F6, even across various excitation wavelengths, suggests a fundamental limitation in their ability to absorb and emit light effectively. In contrast, samples F2, F4, and F5, synthesized with the presence of NaBH_4_ and under optimized conditions, displayed fluorescence, underscoring the importance of using a strong reducing agent and carefully controlled synthesis parameters to achieve the desired fluorescent properties.

TEM imaging highlighted significant morphological differences among the samples. Nonfluorescent formulations F1, F3, and F6 (refer to Figure S1a–c) displayed heterogeneous structures and large agglomerates, deviating from the well-defined nanosheet morphology seen in fluorescent samples. This structural heterogeneity is likely due to the absence of NaBH_4_, which limited the reduction and coordination of PVP with Gd atoms and hindered the formation of uniform nanosheets. Additionally, lower PVP content (3% in F1) and extended ultrasonication (in F3) led to bulkier, less stable structures that further compromised fluorescence.

The role of NaBH_4_ as a potent reducing agent appears critical;^42−44^ in its absence, PVP alone, while acting as a mild reducer, cannot fully drive the reduction process needed to form fluorescent Gd-PVP nanosheets. This incomplete reduction likely results in a mix of partially reduced or unreacted Gd precursors, impeding the formation of the necessary structures for efficient fluorescence. A lack of uniform molecular arrangement in F1, F3, and F6 may disrupt the electronic structure, affecting fluorescence by creating nonradiative relaxation pathways that dissipate energy as heat rather than light. Disordered or amorphous regions within these samples may prevent the formation of structured energy states, thereby hindering fluorescence.

Well-defined morphology is often associated with ordered structures and specific nano- or microscale features, contributing to the optical properties of materials. In the case of F1, F3, and F6, a less-defined morphology, possibly due to aggregation or particle agglomeration, may limit surface area and fluorescence potential. This observation is further supported by the ζ-potential and size stability data, which indicate a tendency for these formulations to aggregate, thereby reducing their effective stability and fluorescence efficiency (refer to Table S3). Morphological irregularities, such as uneven or rough surfaces, could scatter rather than emit light, further reducing observable fluorescence.

Despite the lack of fluorescence, PVP played a key role across all samples by controlling the size and stabilizing nanostructures, ensuring nanosheet formation. However, the nonfluorescent samples (F1, F3, and F6) exhibited less defined nanosheet structures, as shown in Figure S1.

Of the fluorescent samples, the one with the peak excitation wavelengths had the highest concentration of PVP (6%), suggesting a potential correlation between the two. Increasing the PVP concentration enhanced the fluorescence intensity of Gd-PVP nanosheets, likely due to the formation of more extensive Gd–O–PVP linkages, which facilitated improved light absorption. A 6% PVP concentration appears optimal for forming fluorescent moieties, while lower concentrations may lack sufficient structural support for this integration. Interestingly, ultrasonication time had a minimal effect, with results suggesting that a brief 10 min treatment is adequate to achieve effective dispersion without excessive agglomeration or fluorescent moiety degradation. Additionally, excitation wavelengths below 300 nm indicate limitations in the current formulation’s light absorption efficiency, potentially due to the absence of a strong reducing agent. PVP, serving as both a capping or stabilizing agent and a mild reducing agent,^45,46^ likely facilitated the formation of fluorescent groups by coordinating the arrangement of metal and polymer components. In contrast, lower PVP concentrations may not offer adequate structural support or interaction specificity, potentially impeding this formation. This effect is especially noticeable with excitation wavelengths below 300 nm, where both light absorption and fluorescence emission could be insufficient, particularly without a strong reducing agent to assist the process.

Optimization findings indicate that a 10 min low-intensity ultrasound treatment yields the most effective fluorescent composite nanosheets, compared to a 30 min treatment. This shorter duration appears sufficient for achieving the required dispersion and reaction kinetics while avoiding potential drawbacks, such as particle agglomeration or the degradation of fluorescent moieties.

**Figure 3:**
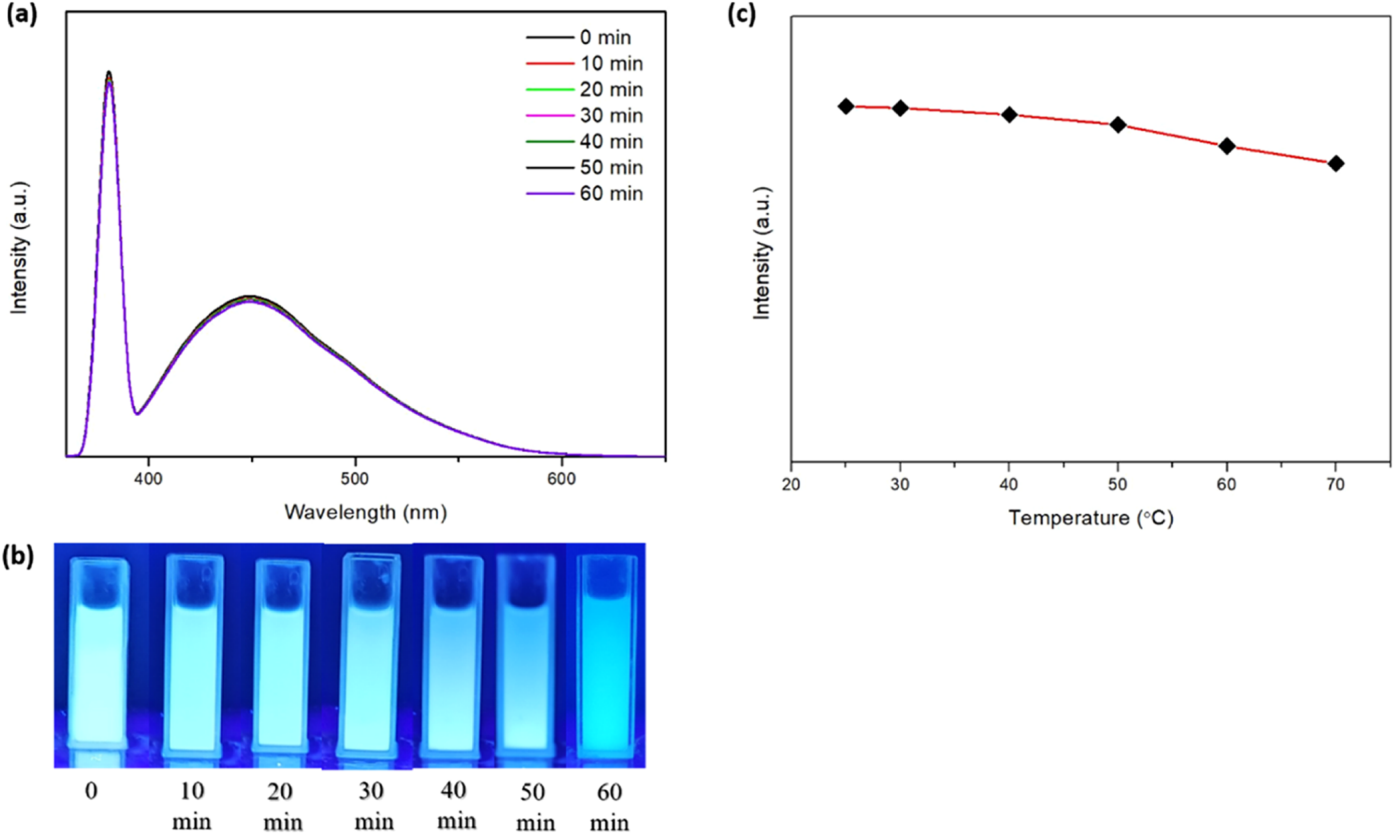
Stability assay of the optimized PVP-Gd composite nanosheets F4 (λex = 380 nm) showing (a) PL emission recorded after exposure to a high-intensity UV lamp (365 nm) for 60 min, (b) UV light-driven images of F4, and (c) PL emission recorded at temperatures ranging from 25 to 70 °C.

Particle stability is critical to the reliability and performance of the detection probe. To evaluate this, we assessed the fluorescent samples’ photostability and thermal stability. Photostability was examined by exposing the samples to UV light for 1 h, while thermal stability was evaluated across a temperature range from 25 to 70 °C. Data analysis involved comparing the PL emission spectra over time to assess photostability (Figure 3a,b). A stable fluorescence intensity indicated good UV degradation resistance, whereas a significant decrease suggested photobleaching. For heat stability, a line graph was used to track the changes in fluorescence intensity at different temperatures (Figure 3c), with minimal fluctuations indicating good heat tolerance of the material. Among the tested samples, F4 exhibited the least photobleaching, maintaining a consistent fluorescence intensity throughout the 1 h exposure period. Conversely, sample F2 exhibited fluctuations and a significant decrease in fluorescence intensity after 1 h of UV light exposure (refer to Figure S5a) indicating susceptibility to photobleaching. Sample F5 displayed less photobleaching over 1 h (refer to Figure S5b) compared to F2, but still performed less favorably than F4.

Moreover, the thermal stability results further confirm the robustness of the optimized PVP-Gd composite nanosheets (F4). As anticipated, the fluorescence intensity of sample F4 remained relatively stable, with only a slight decrease observed as the temperature increased from 50 to 70 °C (see Figure 3c). The slight decrease in fluorescence intensity at higher temperatures is likely due to the inherent thermal sensitivity of the fluorescent agent. However, the relatively stable behavior indicates that the nanosheets can withstand moderate temperature fluctuations without significant loss of fluorescence. The stability of the PVP-Gd composite nanosheets is a result of a synergistic interaction between the inherent properties of the PVP polymer and its strong interactions with the nanosheet surface. PVP, a hydrophilic, biocompatible polymer, plays a crucial role in stabilizing the composite structure through both physical and chemical bonding. The strong binding between PVP and the material’s surface forms a protective “sandwich-like” structure (Gd–O–PVP composite), where PVP molecules surround and shield the active nanosheet components, offering enhanced stability. As previously mentioned, PVP plays a key role in protecting the fluorescent agent bonds, ensuring that the agent remains intact and prevents degradation under both UV and thermal conditions. The tight adhesion of PVP^46^ prevents the degradation of the fluorescent agent upon UV exposure, effectively reducing photobleaching and maintaining fluorescence intensity. When exposed to UV light for 1 h, a higher concentration of PVP offers enhanced protection by acting as a physical shield against high-energy UV radiation, reducing the potential for photodegradation. Moreover, PVP’s antioxidant properties^47^ further contribute to the nanocomposite’s stability by increasing its ability to scavenge reactive oxygen species (ROS) generated during UV exposure. The tightly bound PVP molecules form a protective layer that minimizes the impact of ROS, maintaining the fluorescence of the agent. Additionally, the strong PVP binding ensures the polymer remains firmly attached, preventing the dissociation or disintegration of the composite material even under elevated temperatures. This combination of protective mechanisms—physical shielding, ROS scavenging, and structural stabilization—enhances both the photostability and thermal stability of the PVP-Gd composite, ensuring its integrity under stress and supporting its potential for various applications.

Achieving an optimal balance between PVP concentration and ultrasound treatment proved essential for stability and functionality. As evident in F4, a 10 min ultrasound treatment with a 6% PVP concentration effectively mitigates photobleaching while maintaining uniform dispersion and consistent fluorescence across the nanosheets. This balance likely prevents excessive aggregation, which could diminish fluorescence, while maintaining structural integrity and enhancing the nanosheets’ resistance to both UV and heat-induced degradation.

A comprehensive analysis of DLS, PL, ZP, TEM, reaction yield, and quantum yield data revealed that formulation F4 is the optimal choice for 2D PVP-Gd composite nanosheets. Its superior photostability ensures consistent fluorescence intensity over time, making it a promising candidate for sensor applications.

### 3.3 Formation of Fluorescent PVP-Embedded Gd Composite Nanosheets

The interfacial bonding performance of metal–polymer composite nanomaterials, exemplified by the PVP-Gd_2_O_3_ system, greatly depends on the chemical bonding between the metal oxide (Gd_2_O_3_) and polymer (PVP). These materials constitute a specific class formed through the self-assembly of metal ions and organic ligands, which are molecules possessing functional groups capable of binding to metals, facilitated by coordination bonds.^37,48,49^ The resulting structure is characterized by a network of interconnected metal centers bridged by organic ligands.

Notably, the optimal sample was achieved by employing the highest concentration of PVP (6 wt %) coupled with shorter LIUF exposure times (10 min). In particular, the successful synthesis of the composite nanosheets relies on a delicate interplay between the coordination chemistry of Gd^3+^ ions and the functional groups present in PVP. In this case, the defects or structural changes caused by the reduction process in Gd_2_O_3_ might introduce new energy levels within the material.^50^ These altered energy levels could be responsible for the observed fluorescence in the final PVP-Gd composite nanosheets. Crucially, PVP, while not directly involved in the reduction itself, acts as a stabilizer and capping agent for the Gd_2_O_3_ nanoparticles during synthesis.^38^ This means it prevents uncontrolled growth and aggregation, allowing for the formation of uniform nanosheets with a high surface area. The high surface area is important because it provides more sites for the defects or structural changes to occur, potentially enhancing the overall fluorescence intensity. Ethanol can also help to dissolve PVP and NaBH_4_, facilitating the reduction process. During the reaction, the Gd_2_O_3_–PVP complex likely undergoes self-assembly processes driven by interactions between Gd_2_O_3_ particles, PVP chains, and potentially the introduced fluorescent moieties. This self-assembly leads to the formation of two-dimensional sheet-like structures, resulting in the final fluorescent PVP-embedded Gd composite nanosheets.

The proposed mechanism for the formation of fluorescent 2D PVP-Gd composite NSs involves the unique chemical interactions between the Gd ions in Gd_2_O_3_ and the functional groups in PVP. In Gd_2_O_3_, the d and s orbitals of the Gd ion are vacant, theoretically allowing for the attachment of up to 12 ligands to the central Gd ion through coordinate (dative) bonds due to its 6 empty orbitals. However, the total coordination number of the Gd^3+^ ion typically ranges from 8 to 9, influenced by the steric repulsion of polymers. Consequently, the oxygen atoms of PVP can react with Gd ions, forming Gd–O–C bonds with the carbonyl groups in PVP. Through a novel synergy between PVP and Gd, it has been established that this rare earth metal shows a preference for bonding at imperfectly coordinated Gd sites due to active donor–acceptor interactions. This suggests that the disorder in the Gd ion arrangement in the used precursor (Gd_2_O_3_) might be beneficial for binding with PVP molecules. The variability in the coordination environment around Gd ions provides multiple potential binding sites for PVP, leading to a stable adsorption structure of the nanosheets.

The detailed understanding of the interfacial reactivity and bonding characteristics of Gd-PVP composite materials is shown in the steps below:

**Step 1: Dispersion and Potential Reduction**

The process begins with the breakdown of pre-existing Gd_2_O_3_ into individual Gd^3+^ ions and oxygen ions (O^2–^), followed by NaBH_4_ acts as a reducing agent.^51^

The proposed overall reaction is as below:
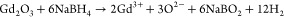


**Step 2: Formation of Gd–O–C Bonds (Key Step for Nanosheet Formation):**

In this step, PVP molecules interact with the Gd^3+^ ions, allowing the Gd^3+^ ions to coordinate with PVP molecules and form complexes where Gd is surrounded by PVP ligands. The oxygen atoms in the carbonyl groups (–C=O) of PVP establish coordinate bonds with the Gd^3+^ ions, contributing to the stability of the resulting Gd-PVP complexes. The lone electron pairs on the oxygen atoms of PVP’s carbonyl groups form coordinate bonds (dative bonds) with the vacant orbitals of Gd^3+^ ions. This bonding is facilitated by the attraction between the positively charged Gd ions and the negatively charged oxygen atoms, creating a donor–acceptor interaction. As the concentration of PVP increases, there is a higher likelihood of Gd^3+^ ions encountering PVP molecules, which promotes greater formation of Gd–O–C bonds.^39,52,53^
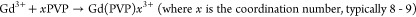


**Step 3: Network Formation, Stability, and Fluorescence Enhancement:**

The steric bulk of PVP chains limits the number of PVP molecules that can bond to a single Gd^3+^ ion, typically resulting in a coordination number of 8 to 9. With more PVP available, a network of Gd-PVP complexes forms due to the multiple potential binding sites created by the variability in Gd ion arrangement within Gd_2_O_3_ (imperfect coordination) and the steric effects of PVP chains. The repulsion between PVP chains prevents aggregation and maintains a well-dispersed nanosheet structure. Strong interfacial bonding between Gd and PVP likely contributes to the enhanced fluorescence properties observed in the final 2D PVP-Gd composite NSs.
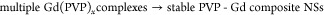
Overall, the cooperative interplay between Gd^3+^ seeking electron donors, PVP offering suitable bonding sites, and the spatial influence of the PVP chains (steric effects) leads to the self-assembly of Gd-PVP complexes into a sheet-like nanosheet structure. This structure is responsible for the final properties of the fluorescent Gd-PVP composite nanosheets.

### 3.4 Structural Characterization

**Figure 4:**
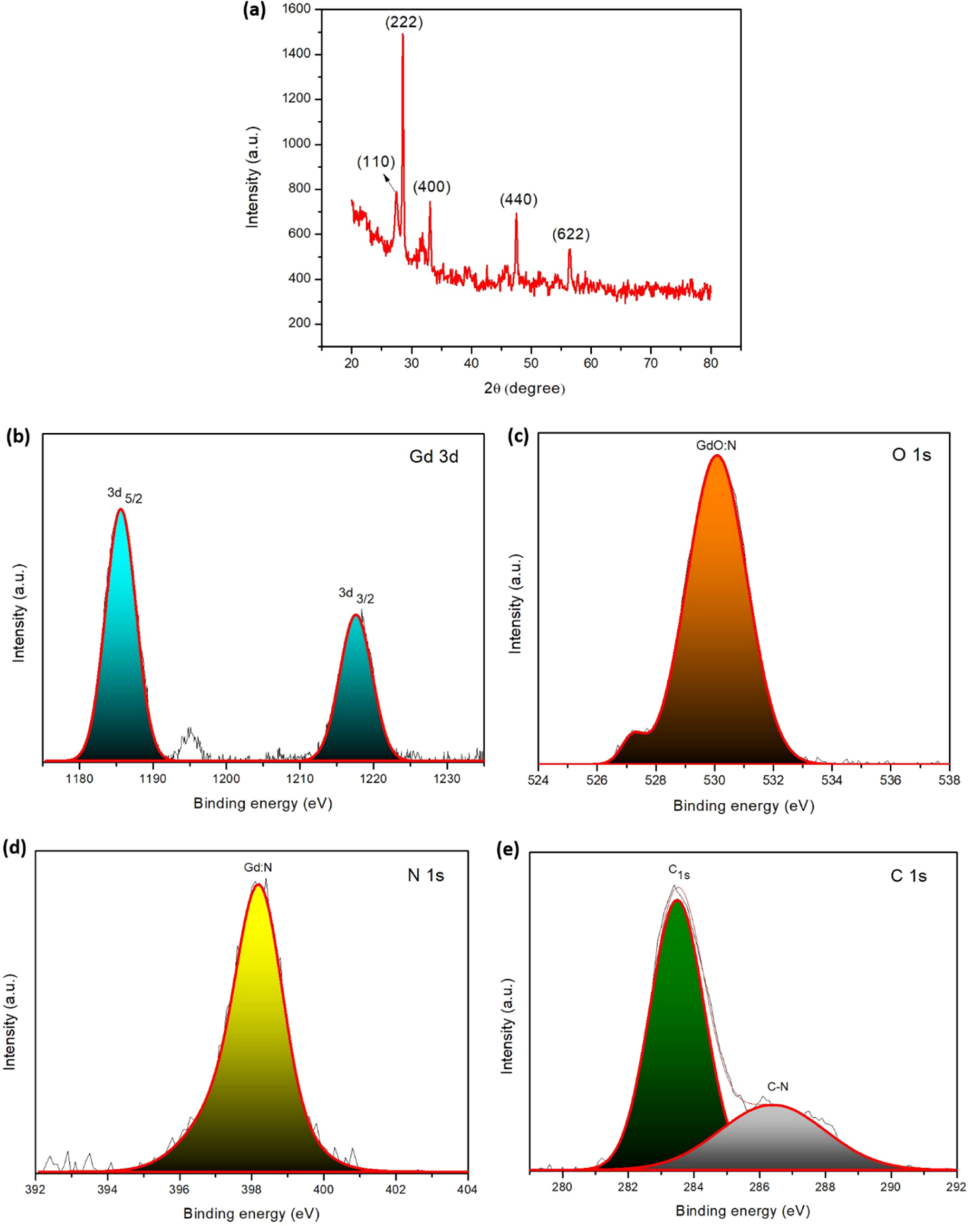
(a) XRD pattern and XPS spectra of (b) Gd 3d, (c) O 1s, (d) N 1s, and (e) C 1s for 2D PVP-Gd composite nanosheets.

When a polymer is complexed with a salt or metal oxide, the crystallinity of the polymeric host can be disrupted by the introduction of impurities. This can lead to changes in the XRD pattern, such as the broadening of peaks or the appearance of new peaks. The XRD patterns of the 2D PVP-Gd composite NSs (Figure 4a) exhibit diffraction peaks corresponding to crystalline phases of the Gd-PVP composite. The presence of a distinctive (110) peak in the XRD pattern likely indicates the formation of a new crystalline phase between Gd and PVP, signifying the successful incorporation of Gd into the PVP matrix. The enhanced intensity of the (222) peak compared to other expected peaks further supports the formation of a dominant new crystalline phase, likely involving Gd, confirming the presence of Gd in the composite. The presence of well-defined peaks at (400), (440), and (622) in the XRD pattern indicates the excellent crystallinity of the gadolinium oxide (Gd_2_O_3_) phase. These peaks match the cubic bixbyite body-centered structure of Gd_2_O_3_ (space group I_a3_ No. 206) reported in JCPDS file number 86–2477.^32,48^

As reported in the literature^54−56^ the XRD pattern of pure PVP exhibits distinctive features indicative of its amorphous and semicrystalline nature. A broad peak observed around 2θ ≈ 18–22° is typically associated with the amorphous nature of pure PVP. This peak suggests a slight degree of short-range order or crystallinity within the polymer matrix, which can vary depending on the molecular weight of PVP and the processing conditions. The position of this peak may shift slightly under different conditions, indicating the influence of the polymer’s molecular structure on its degree of order. In addition to this broad peak, weak diffraction peaks between 2θ ≈ 20–30° can be observed in some instances. These peaks are indicative of semicrystalline regions within PVP, suggesting partial order either within the polymer chains themselves or between adjacent chains. However, these peaks are relatively weak and broad compared to those seen in highly crystalline materials, reflecting the low degree of crystallinity present in pure PVP. A wide diffuse feature typically appears in the range of 2θ ≈ 12–15°, which is characteristic of amorphous polymers like PVP. This feature represents the disordered regions within the material, further supporting the predominantly amorphous structure of pure PVP.

Notably, the observed shifts in peak positions compared to pure PVP indicate potential interactions at the atomic level, altering the crystal lattice structure. These peak shifts can be attributed to lattice strain or alterations in lattice parameters induced by the composite formation, indicating the effective integration of Gd atoms within the PVP matrix. The sharpness and intensity of the diffraction peaks offer insights into the crystallinity of the composite. Unlike crystalline materials, which exhibit sharp, well-defined peaks, pure PVP’s XRD pattern lacks distinct diffraction signals. In contrast, the XRD pattern of the Gd-PVP composite NSs displays more prominent peaks corresponding to the crystalline phases of gadolinium oxide (Gd_2_O_3_), indicating the successful incorporation of the inorganic phase into the polymer matrix.

XPS was utilized to investigate the elemental composition and surface chemical bonding states of the optimized 2D PVP-Gd composite NSs. During analysis, a pass energy of 80 eV was employed for the survey spectra. The XPS spectra confirm the presence of Gd, O, N, and C (Figure 4b–e).^48,57,45^ The presence of Gd is evident from peaks corresponding to its characteristic electron energy levels, such as Gd 4d and Gd 3d. Similarly, C 1s and N 1s peaks confirm the presence of PVP within the composite. Interestingly, the C 1s (C–C, C–NH_2_, C–O), N 1s (Gd:N) and O 1s (GdO:N) peaks exhibit shifts compared to their positions in pure PVP.^39,45^ This shift suggests changes in the chemical environment surrounding these atoms, potentially due to the formation of coordination bonds or other interactions between Gd and functional groups present in the PVP molecule.

### 3.5 Evaluation of Fluorescence Sensor Performance

**Figure 5:**
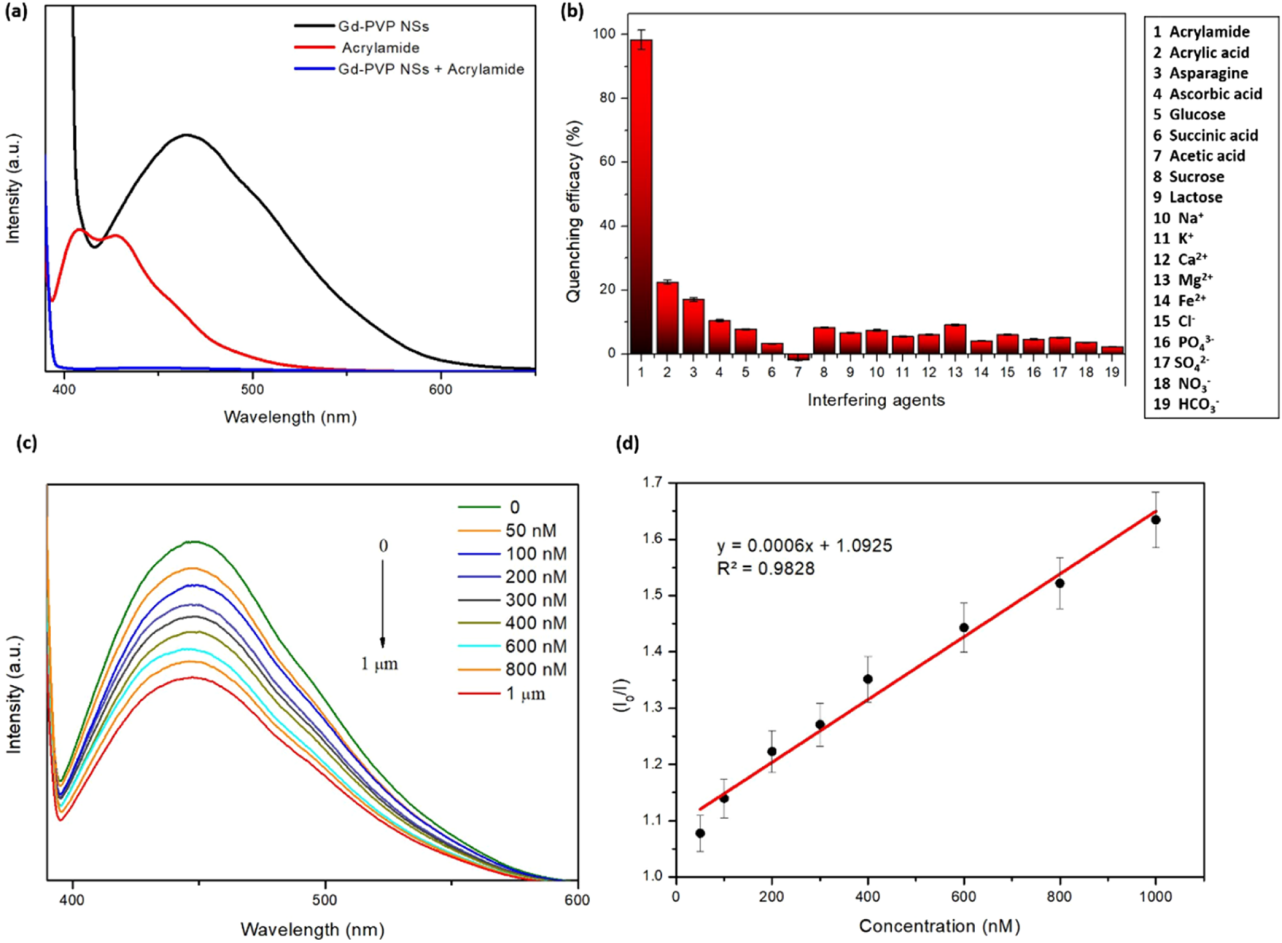
Acrylamide (AM) detection assay (λ_ex_ = 380 nm). (a) fluorescence response of the optimized PVP-Gd composite nanosheets (F1) toward AM, (b) %fluorescence quenching efficacy of the optimized PVP-Gd composite nanosheets (F1) toward AM, and a variety of interfering agents (0.5 μM each in DI water)., (c) emission spectra with the increasing concentration of AM, and (d) linear fitting relationship between the PL intensity and AM concentration.

To confirm the sensor’s selectivity specifically for AM, various structural analogs of AM (including acrylic acid, ascorbic acid, succinic acid, acetic acid, glucose, sucrose, lactose, and asparagine) as well as common ions and anions found in food were introduced for observation (refer to Figure 5). A low concentration of AM could induce significant fluorescence quenching in 2D PVP-Gd composite NSs (Figure 5a), resulting in a response intensity greater than that caused by other interfering substances (Figure 5b). No significant impact on the detection of AM was observed when introducing high concentrations of interfering ions or the diluting solvent, indicating the sensor’s high selectivity. Common interfering acids employed in this study (succinic, and acetic) had minimal impact, likely due to differences in their “–CHO” group activity compared to AM, which is more readily detected by the sensor (Figure 5b). Fluorescence changes induced by AM were significantly higher compared to those caused by the other six substances at an equivalent concentration (0.4 mg/L) of potential interference. Hence, the method exhibited excellent specificity. It can be postulated that AM molecules possess functional groups like the amide group (−C=O–NH_2_) that can form hydrogen bonds with suitable groups on the nanosheet surface (e.g., −OH groups in PVP).^39,58^ This hydrogen bonding could potentially induce alterations in the conformation of surrounding molecules, thereby influencing the electronic properties of the nanosheet and consequently affecting its fluorescence intensity.

To assess the assay’s sensitivity, the fluorescence response of the detection system was monitored following the addition of varying AM concentrations (50, 100, 200, 300, 400, 600, 800, and 1000 nM) under the established optimal reaction conditions (Figure 5c). A standard curve (Figure 5d) using the logarithm of AM concentration on the *x*-axis and the change in fluorescence (*I*_0_/*I*) of the detection system on the *y*-axis was created to quantify the assay’s sensitivity. As the AM concentration increased within the range of 50–1000 nM, the fluorescence intensity proportionally decreased. This linear relationship is confirmed by the equation: *y* = 0.0006*x* + 1.0925; (*R*^2^ = 0.9828). The limit of detection (LOD) of the produced sensor for AM was calculated to be 9.4 nM using the equation LOD = 3.3σ/κ, where σ represents the standard deviation from the blank measurement and κ represents the slope of the linear calibration curve. Upon comparing the method developed in this study with other AM detection techniques (referenced in Table 1),^59−65^ it is evident that the quenching-based fluorescent tracer proposed here offers a lower LOD and a broader linear range than methods such as capillary electrophoresis method (CE), HPLC-MS, and enzyme-linked immunosorbent assay (ELISA). This suggests that the AM assay established in this study offers superior sensitivity.

**Table 1:** Comparison of the Results of Different Methods for Detecting Acrylamide

sensor type	detection range	LOD	references
fluorescence nanocomposites	0–1 μM	9.4 nM	this work
HPLC-MS	79–710 nM	30 nM	[^59^]
SERS	70–140 nM	28 nM	[^60^]
quartz crystal microbalance	0.5–10 μM	0.39 μM	[^61^]
GC-MS with bromination	0–18,000 μM	3.8 μM	[^62^]
AA-GSH-Au NPs-TMB	0.5–175 μM	0.16 μM	[^63^]
ELISA	230–5600 μg/L	89 μg/L	[^64^]
CE	2.5–40 mg/L	0.32–0.56 mg/L	[^65^]
filter paper-based SANC	0.1 nM–50 μM	0.02 nM	[^66^]
PEC nanocomposites	10^–1^ M–2.5 × 10^–9^	2.147 × 10^–9^ M	[^67^]

**Figure 6:**
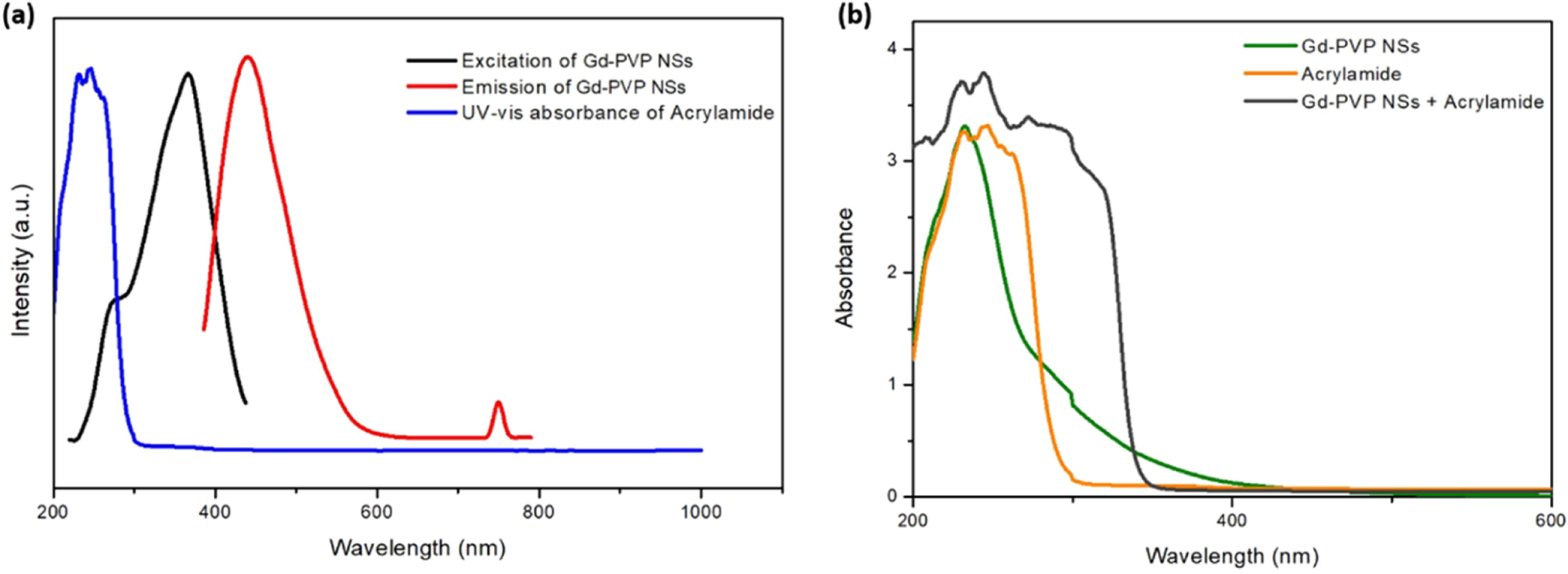
Interaction of the optimized PVP-Gd composite nanosheets (F1) and acrylamide (a) excitation and emission spectra for the PVP-Gd composite nanosheets and UV–vis absorption spectra for acrylamide alone and (b) UV–vis absorption spectra for acrylamide, bare PVP-Gd composite nanosheets, and PVP-Gd composite nanosheets after addition of acrylamide.

The detection system underwent further characterization steps, employing UV–visible absorption spectroscopy (refer to Figure 6b) and ZP measurements (Figure S6). After incubating with AM, the 2D PVP-Gd composite NSs were rinsed with DI water, gradually removing unbound AM. The emergence of several distinct absorption bands strongly suggests successful AM coupling to the nanosheets. Notably, individual nanosheets displayed an absorption peak of around 232 nm. Interestingly, upon binding with the analyte, a clear additional band emerged at approximately 245 nm. This distinct spectral signature confirms the association between the nanosensor and the target molecule (AM) after their interaction. Introducing AM to the 2D Gd-PVP composite NSs caused a significant decrease in the ZP values, down to 2.75 mV (Figure S6b). This shift suggests a complex formation between AM and the sensor. The reduced ZP indicates AM’s ability to neutralize the surface charge of the 2D Gd-PVP composite NSs, potentially forming a nonfluorescent 2D Gd-PVP composite NSs-AM complex in the ground state.

### 3.6 Application in Real Samples by the Current Approach

The feasibility and accuracy of the 2D PVP-Gd composite nanosheets sensor for detecting acrylamide (AM) in real food samples were successfully demonstrated in this study (Table 2). The method was validated by analyzing potato chip and fried biscuit samples, with the extracts appropriately diluted in a 0.1 M PBS solution containing AM at pH 7.0. The calibration curve obtained from the real food matrix showed a slope of 1.15 ± 0.003 and an intercept of 0.03 ± 0.018. The small standard deviations associated with both the slope and intercept indicate that the calibration model is precise and reproducible. This suggests that the sensor provides consistent responses to AM concentrations, even when subjected to the complexities of real food matrices. The low standard deviation of the slope (±0.003) and intercept (±0.018) reflects the reliability of the method, which is essential for ensuring the accuracy of the sensor in practical applications.

**Table 2:** Analytical Results of AM Using PVP-Gd Composite NSs in Real Samples (*n* = 3)^a^

samples	spiked (nM)	found (nM)	recovery (%)	RSD (%) (*n* = 3)
potato chips	100	100.44	104.00	±2.41
	300	300.61	108.72	±1.37
	600	600.50	105.53	±2.18
biscuits	100	98.30	98.78	±0.47
	300	296.16	97.41	±0.61
	600	599.66	98.72	±0.54
bread	100	99.11	99.24	±0.48
	300	300.52	105.77	±1.51
	600	600.71	109.37	±2.47

a Values reported are averages of 3 independent measurements for each sample; RSD: relative standard deviation.

In addition to the calibration data, the matrix effect was quantified by comparing the matrix-matched calibration curve to the standard calibration curve (generated using a matrix-free solution). The matrix effect was found to be 12%, indicating moderate interference from the food matrix components, such as fats, sugars, and other organic compounds in the potato chips and fried biscuits. However, the matrix effect observed in this study did not significantly affect the sensor’s performance, as evidenced by the good recovery rates obtained in the real samples, ranging from 96.0 to 103.0% for potato chips and 97.6 to 99.6% for fried biscuits. The 12% matrix effect suggests that while there is some influence from the matrix, the sensor’s overall performance remains robust. The method’s low relative standard deviations (RSD) in both the potato chips (±1.54%) and fried biscuits (±1.11%) further support the reliability of the sensor, even in the presence of matrix interference. These findings highlight the sensor’s suitability for acrylamide detection in real-world food products despite the potential challenges posed by complex matrices.

### 3.7 Fluorescence Quenching Mechanism of 2D PVP-Gd Composite NSs

The fluorescence quenching observed in the 2D PVP-Gd NSs-AM system can be primarily attributed to photoinduced electron transfer (PET),^68^ supported by collisional quenching.^69^ PET is the dominant mechanism due to its nonradiative energy transfer nature.^70^ Here, the AM (electron acceptor) having a lower electron affinity compared to the excited NSs (donor) could facilitate electron transfer from the NSs to AM. This electron transfer might contribute to the decrease in the fluorescence of the 2D PVP-Gd composite NSs. In this scenario, a direct transfer of an electron occurs from the excited donor (NSs) to the acceptor (AM) in the ground state. This electron transfer may quench the excited state of the donor, preventing it from emitting a photon, thus quenching its fluorescence.

In contrast, collisional quenching, which relies on the diffusion and collision of AM molecules with the excited nanosheets, provides additional quenching but is secondary in efficiency compared to electron transfer. Upon the addition of AM, the ZP of the PVP-Gd composite NSs decreases significantly (refer to Figure S6b), indicating the adsorption of AM molecules onto the nanosheets’ surface. This adsorption leads to the neutralization of the surface charge, reducing the electrostatic repulsion between nanosheets and promoting a more aggregated state. This change in surface charge is a clear sign of the interaction between AM and the PVP-Gd composite NSs, which is crucial for the quenching process. When excited, the NSs possess higher energy electrons. If the AM molecule collides with an excited NSs in close proximity, the excited electron in the NSs can transfer its energy to the AM molecules, promoting it to a higher energy level. This transferred energy is then dissipated by the AM molecule through nonradiative pathways, such as vibrations or heat, resulting in a significant decrease in the fluorescence intensity of the NSs (refer to Figure 5a).

The energy transfer is possible because, as shown in Figure 6a, the light absorption patterns of the nanosheets and AM overlap. This overlap allows the AM molecules to absorb the light energy emitted by the excited nanosheets, instead of the energy being released as fluorescence. The presence of AM further strengthens this effect. The spectral overlap facilitates efficient energy transfer processes central to the quenching mechanism.

The addition of AM affects the light absorption properties of the nanosheets (Figure 6b). This suggests a strong interaction between the AM molecules and the nanosheets. The overlap between the light absorption patterns of the AM and the nanosheets indicates that the AM molecules can exchange energy with the excited nanosheets. This energy exchange reduces the fluorescence intensity significantly. This interaction between the AM and the nanosheets changes how light interacts with them, affecting their overall light properties. The changes in light absorption also suggest that the structure of the nanosheets themselves is slightly altered by the AM, which is consistent with mechanisms involving energy transfer between the molecules.^71^ Additionally, the close proximity caused by the AM interaction with the nanosheets facilitates frequent collisions. These collisions can directly deactivate the excited state of the nanosheets without light emission, effectively quenching their fluorescence through a mechanism called collisional quenching, as discussed earlier. Overall, the changes in the UV–vis spectra support the idea that the interaction with AM disrupts the excited state of the nanosheets, leading to a decrease in fluorescence.

As a sensor probe, the PVP-Gd composite nanosheet offers a unique combination of properties that contribute to its selectivity and sensitivity toward acrylamide. PVP, a versatile polymer, provides a consistent matrix for analyte adsorption, while Gd ions enhance the sensor’s optical properties. The synergistic interaction between PVP and Gd results in efficient fluorescence quenching, particularly through energy transfer and heavy atom effects. The biocompatible, nontoxic PVP polymer, with its hydrophilic −N–C=O carbonyl amide groups and hydrophobic C–C polymer chain, effectively interacts with analytes due to its proton-accepting nature, enhancing sensor sensitivity and stability.^72^ Furthermore, PVP interacts effectively with acrylamide through hydrogen bonding and van der Waals forces, enhancing its ability to detect acrylamide. Acrylamide, being both a strong hydrogen bond donor and acceptor, interacts with highly electronegative atoms such as oxygen (O) and nitrogen (N),^73^ which are present in the PVP structure. By treating the Gd substrate with PVP, chemical enhancement is achieved, as the surface-modified composite nanosheets form strong hydrogen bond complexes with acrylamide in the sample. These interactions are key to the sensor’s ability to detect acrylamide specifically while minimizing interference from other molecules. Gd’s ability to exhibit luminescent behavior, converting near-infrared light to visible or ultraviolet light, contributes to the sensor’s unique optical properties. The interaction between the Gd ions and acrylamide also plays a significant role in fluorescence quenching through both collisional interactions and energy transfer processes. The heavy atom effect, promoted by Gd, increases spin–orbit coupling, leading to nonradiative decay and efficient quenching. Additionally, the two-dimensional structure of the nanosheets provides a large surface area, facilitating rapid adsorption of acrylamide molecules, which further enhances the detection sensitivity. In a recent study, Vashistha et al. reported^74^ the successful synthesis of PVP-capped, rod-shaped europium (Eu^3+^)-doped gadolinium oxide (Gd_2_O_3_) nanoparticles (PVP@Gd_2_O_3_:Eu^3+^ NPs), which exhibited strong fluorescence emission in aqueous solutions. The produced PVP-Gd composite NSs leverage the combined advantages of PVP’s stability and Gd’s optical properties to create a highly efficient and selective acrylamide sensor without the need for complex modifications seen in other nanomaterial-based sensors.

In conclusion, this study introduces an innovative organic–inorganic nanocomposite-based sensor tailored for sensitive AM detection. By systematically investigating the effects of PVP concentration and low-intensity ultrasound treatment times, we optimized the synthesis of metal–polymer composite nanosheets to enhance efficiency and reduce energy consumption. The fluorescence observed in the synthesized nanocomposites arises from the intrinsic properties of the materials and their interactions, achieving a notably high quantum yield of 45.01%. This efficient, nontraditional fluorescence-inducing method highlights PVP’s multifaceted role as a stabilizer, dispersant, and growth modifier, crucial in creating well-defined, stable nanosheets optimized for AM detection. The resulting PVP-Gd composite sensor exhibits a wide detection range and a low detection limit (9.4 nM) with successful AM detection in real food samples with high recovery rates. The sensor offers a rapid response, making it ideal for real-time monitoring in food safety and environmental applications. This method is both economical and highly scalable by utilizing commercially available, cost-effective chemicals, combined with LIFU treatment and a low synthesis temperature of 150 °C.

## Data Availability

The data used to support the findings of this study are included in the article.

## Supporting Information Available

Particle size and particle size stability measurement; optimization of 2D PVP-Gd composite nanosheet preparation; the excitation and emission wavelength values of the nanocomposites; ζ-potential; size increase (%) values after 30 days; reaction yield; and quantum yield of the nanocomposites; TEM images of the nanocomposites; EDS spectra and a table of weight percentages; elemental mapping of the optimized PVP-Gd composite nanosheet; optimization of fluorescence intensity, photostability assay of the nanocomposite; and ζ-potential distributions (PDF)
