# Supplementary material for: Self-Assembled PVP-Gd Composite Nanosheets via Ultrasound Synthesis for Targeted Acrylamide Sensing in Food Safety
Source: J Agric Food Chem. 2025 Feb 11;73(8):4887–903. doi: 10.1021/acs.jafc.4c08460 (PMC11869275; doi:10.1021/acs.jafc.4c08460)
Supplement: Supplementary file 1 — jf4c08460_si_001.pdf [file jf4c08460_si_001.pdf]

Supporting Information

Self-Assembled PVP-Gd Composite Nanosheets via Ultrasound Synthesis for Targeted Acrylamide Sensing in Food Safety

Sahar Pakbaten Toopkanloo[1], Hui-Fen Wu*[1234567]

[1]International PhD Program for Science, National Sun Yat-Sen University, Kaohsiung, 80424, Taiwan

[2]Department of Chemistry, National Sun Yat-Sen University, Kaohsiung, 80424, Taiwan.

[3]School of Pharmacy, College of Pharmacy, Kaohsiung Medical University, Kaohsiung, 807, Taiwan.

[4]School of Medicine, College of Medicine, National Sun Yat-Sen University, Kaohsiung, 80424, Taiwan.

[5]Institute of Medical Science and Technology, National Sun Yat-Sen University, Kaohsiung, 80424, Taiwan.

[6]Institute of Precision Medicine, National Sun Yat-Sen University, Kaohsiung, 80424, Taiwan.

[7]Institute of BioPharmaceutical Science, National Sun Yat-Sen University, Kaohsiung, 80424, Taiwan

*Corresponding author: Prof. Hui-Fen Wu (Department of Chemistry, National Sun Yat-Sen University)

*Email: hwu@faculty.nsysu.edu.tw

Phone: +886-7-5252000-3955; Fax: +886-7-5253909

S1. Particle Size and Particle Size Stability Measurement

The mean particle size (z-average) of the prepared 2D nanocomposites was assessed by dynamic light scattering (DLS) using an ELSZ-2000 instrument (Otsuka Electronic, Japan) at room temperature. All solutions were diluted in DI water before measurement. The particle size stability of the composites was also analyzed after 30 days of storage to assess the impact of preparation conditions on the rate of change in particle size over time. The prepared composites were always stored in darkness at room temperature. Triplicate analyses were run for each sample.

The particle size value change rate caused by a 30-day of storage, given as a percentage, was calculated using Equation (1):

$$\text{Value change rate (\%)} = \frac{[\text{particle size after 30 days (nm)} - \text{initial particle size (nm)}] \times 100}{\text{initial particle size(nm)}}$$

Table S1. Optimization of 2D PVP-Gd composite nanosheet preparation; General Full Factorial Design matrix.

Formulation Code	Runs	X_1 (PVP, wt.%)	X_2 ($NaBH_4$, g)	X_3 (LIFU time, min)
F1	1	3	0	10
F2	2	3	1	30
F3	3	6	0	30
F4	4	6	1	10
F5	5	3	1	10
F6	6	6	0	10

Table S2. The excitation and emission wavelength values of the prepared composites.

Formulation Code	Excitation (nm)	Emission (nm)
F1	-	non-fluorescent
F2	320	390
F3	-	non-fluorescent
F4	380	460
F5	310	397
F6	-	non-fluorescent

Table S3. The zeta potential (ZP), size increase (%) values after 30 days, reaction yield (RY), and quantum yield (QY) of the prepared composites. (n=3)[a]

Formulation Code	ZP (mV)	Size increase (%)	RY (%)	QY (%)
F1	+ 7.26 ± 0.96	50.0	23	non fluorescent
F2	+ 19.81 ± 1.71	39.7	63	25.42
F3	+ 9.32 ± 2.18	48.6	29	non fluorescent
F4	+ 29.86 ± 1.35	24.0	92	45.01
F5	+ 23.21 ± 2.62	28.8	71	29.43
F6	+ 17.14 ± 2.51	32.7	38	non fluorescent

[a] Values reported are averages of 3 independent measurements for each sample.

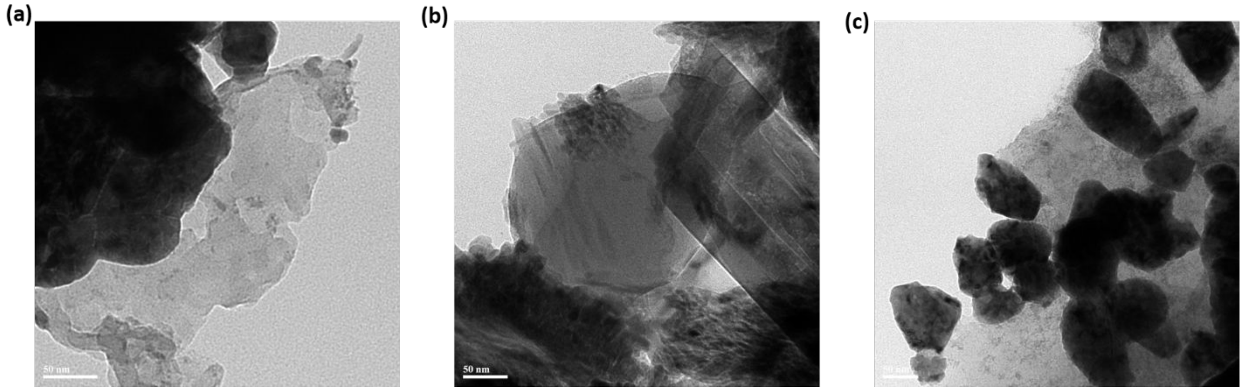

Figure S1. (a–c) TEM images (50 nm scale) of PVP-Gd nanocomposites for F1, F3, and F6, respectively.

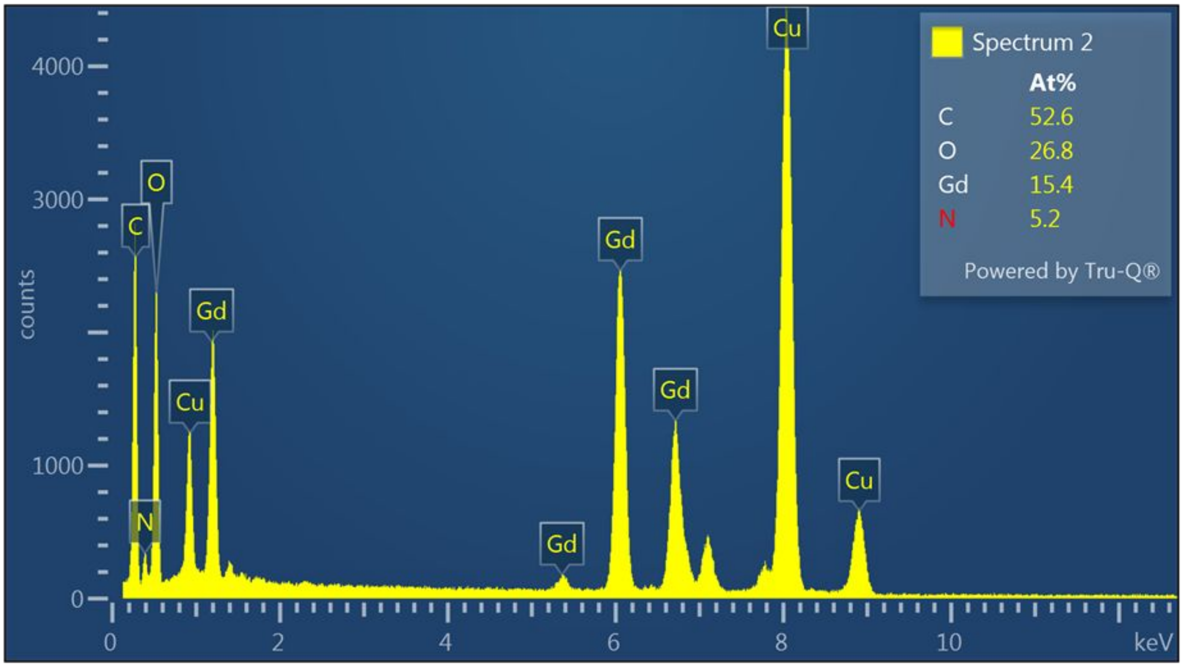

Spectrum 2							
Element	Line Type	k Factor	k Factor type	Absorption Correction	Wt%	Wt% Sigma	Atomic %
C	K series	2.644	Theoretical	1.00	17.77	0.28	52.56
N	K series	3.372	Theoretical	1.00	2.06	0.21	5.22
O	K series	1.947	Theoretical	1.00	12.09	0.19	26.84
Gd	L series	2.258	Theoretical	1.00	68.08	0.34	15.38
Total:					100.00		100.00

Figure S2. The elemental ratios are presented in the EDS pattern and a table showing the weight percentages (wt%) for the optimized PVP-Gd composite nanosheet (F4).

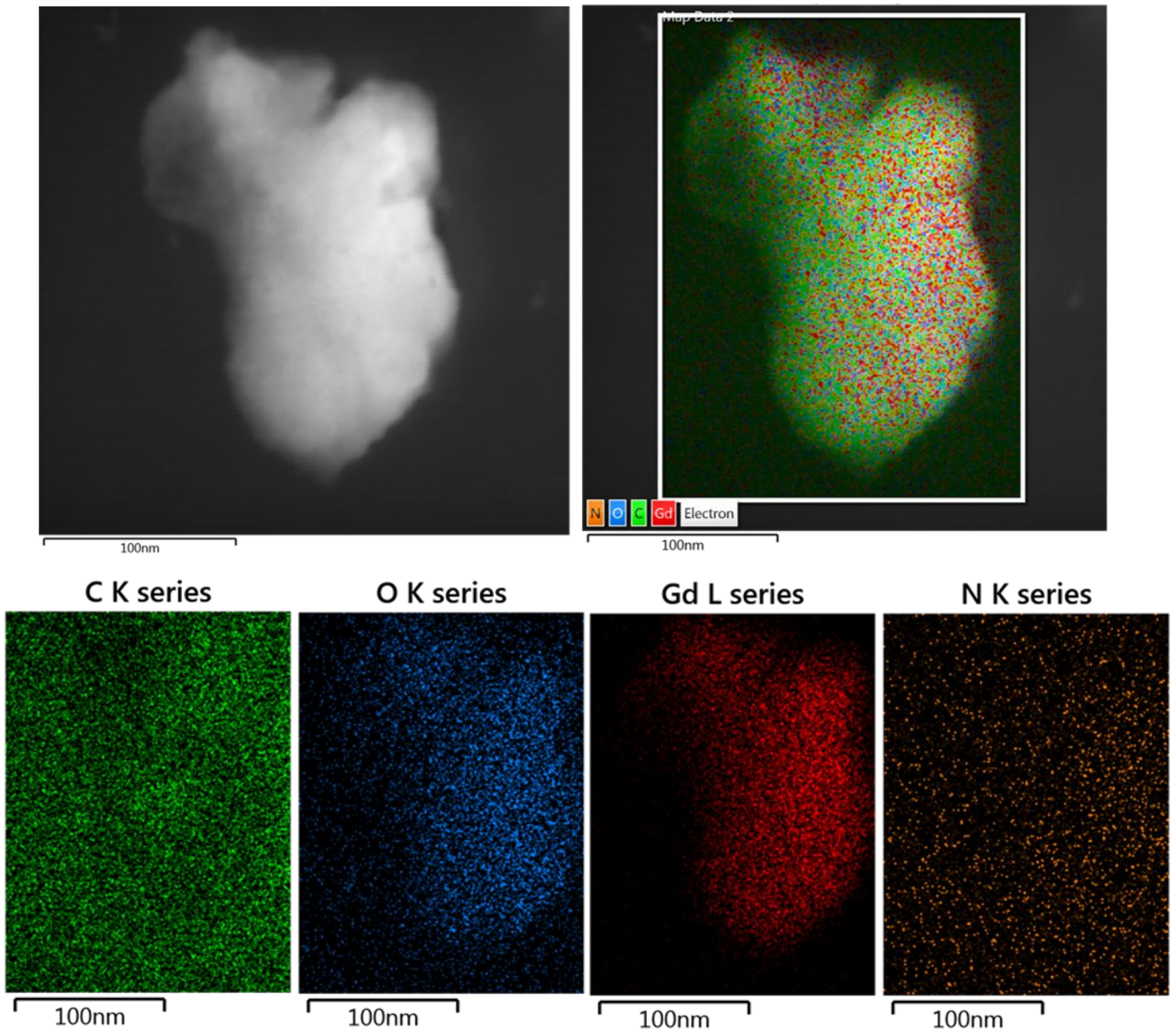

Figure S3. Elemental mapping of the optimized PVP-Gd composite nanosheet (F4) indicates the distribution of key constituents: Carbon (C), Oxygen (O), Gadolinium (Gd), and Nitrogen (N).

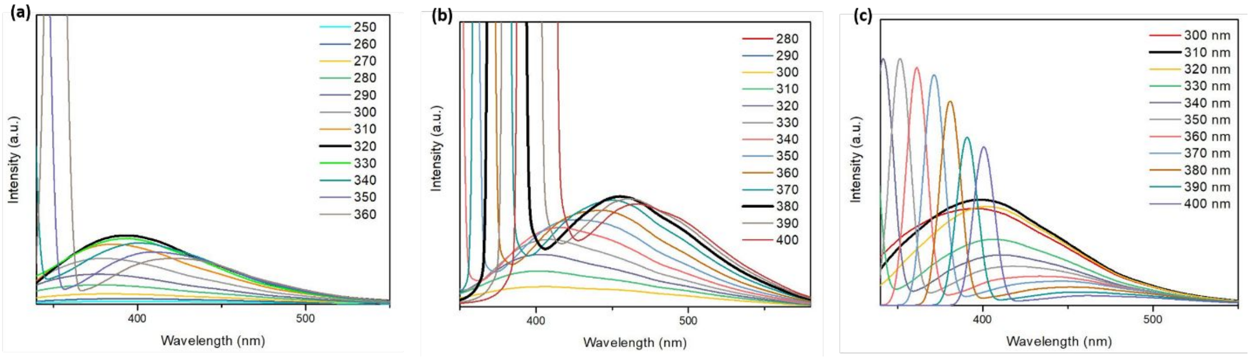

Figure S4. Optimization of fluorescence intensity at various wavelengths (λex = 300 - 400 nm) of (a) F2, (b) F4, and (c) F5.

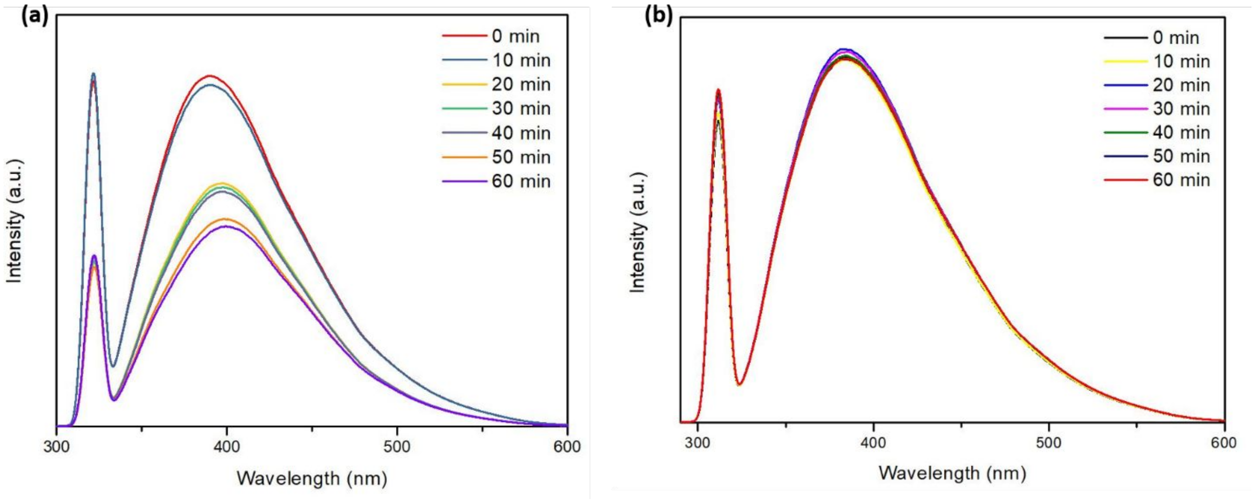

Figure S5. Photostability assay of the 2D PVP-Gd composite nanosheets using a high-intensity UV lamp (365 nm) for 60 minutes (a) recorded PL emission for F2 (λex = 320 nm), and (b) recorded PL emission for F5 (λex = 310 nm).

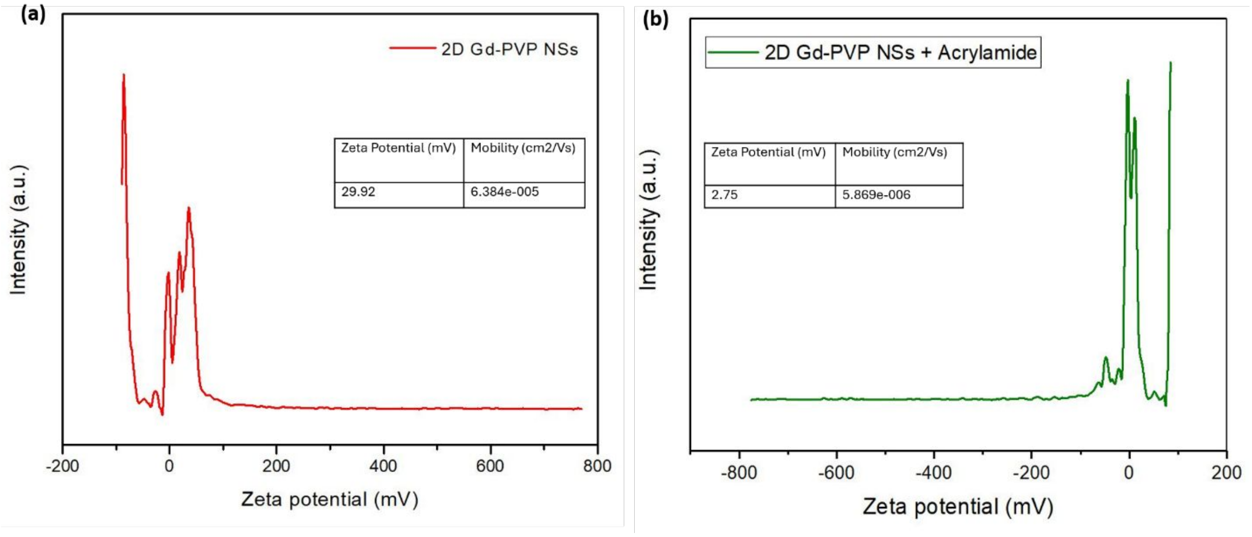

Figure S6. Zeta potential distributions of (a) 2D Gd-PVP composite nanosheets alone, and (b) 2D Gd-PVP composite nanosheets with the acrylamide (AM).
